# Comparative Whole Genome Sequence Analysis and Biological Features of *Clostridioides difficile* Sequence Type 2^‡^

**DOI:** 10.3389/fmicb.2021.651520

**Published:** 2021-07-05

**Authors:** Xingxing Xu, Qiao Bian, Yun Luo, Xiaojun Song, Shan Lin, Huan Chen, Qian Liang, Meixia Wang, Guangyong Ye, Bo Zhu, Liang Chen, Yi-Wei Tang, Xianjun Wang, Dazhi Jin

**Affiliations:** ^1^Department of Clinical Laboratory, Women’s Hospital, Zhejiang University School of Medicine, Hangzhou, China; ^2^School of Laboratory Medicine, Hangzhou Medical College, Hangzhou, China; ^3^Zhejiang Provincial Center for Disease Control and Prevention, Hangzhou, China; ^4^School of Biotechnology and Biomolecular Sciences, University of New South Wales, Sydney, NSW, Australia; ^5^Centre of Laboratory Medicine, Zhejiang Provincial People’s Hospital, People’s Hospital of Hangzhou Medical College, Hangzhou, China; ^6^Key Laboratory of Microorganism Technology and Bioinformatics Research of Zhejiang Province, Hangzhou, China; ^7^NMPA Key Laboratory for Testing and Risk Warning of Pharmaceutical Microbiology, Hangzhou, China; ^8^Center for Discovery and Innovation, Hackensack Meridian Health, Nutley, NJ, Untied States; ^9^Department of Medical Sciences, Hackensack Meridian School of Medicine, Nutley, NJ, Untied States; ^10^Cepheid, Danaher Diagnostic Platform, Shanghai, China; ^11^Department of Clinical Laboratory, Hangzhou First People’s Hospital, Zhejiang University School of Medicine, Hangzhou, China

**Keywords:** *Clostridioides difficile*, whole genome sequencing, ST2, *tcdB*, genomic characteristics

## Abstract

*Clostridioides difficile* sequence type 2 (ST2) has been increasingly recognized as one of the major genotypes in China, while the genomic characteristics and biological phenotypes of Chinese ST2 strains remain to be determined. We used whole-genome sequencing and phylogenetic analysis to investigate the genomic features of 182 ST2 strains, isolated between 2011 and 2017. PCR ribotyping (RT) was performed, and antibiotic resistance, toxin concentration, and sporulation capacity were measured. The core genome Maximum-likelihood phylogenetic analysis showed that ST2 strains were distinctly segregated into two genetically diverse lineages [L1 (67.0% from Northern America) and L2], while L2 further divided into two sub-lineages, SL2a and SL2b (73.5% from China). The 36 virulence-related genes were widely distributed in ST2 genomes, but in which only 11 antibiotic resistance-associated genes were dispersedly found. Among the 25 SL2b sequenced isolates, RT014 (40.0%, *n* = 10) and RT020 (28.0%, *n* = 7) were two main genotypes with no significant difference on antibiotic resistance (χ^2^ = 0.024–2.667, *P* > 0.05). A non-synonymous amino acid substitution was found in *tcdB* (Y1975D) which was specific to SL2b. Although there was no significant difference in sporulation capacity between the two lineages, the average toxin B concentration (5.11 ± 3.20 ng/μL) in SL2b was significantly lower in comparison to those in L1 (10.49 ± 15.82 ng/μL) and SL2a (13.92 ± 2.39 ng/μL) (χ^2^ = 12.30, *P* < 0.05). This study described the genomic characteristics of *C. difficile* ST2, with many virulence loci and few antibiotic resistance elements. The Chinese ST2 strains with the mutation in codon 1975 of the *tcdB* gene clustering in SL2b circulating in China express low toxin B, which may be associated with mild or moderate *C. difficile* infection.

## Introduction

*Clostridioides difficile* is an anaerobic, spore-forming Gram-positive bacillus that is able to colonize and proliferate in the human gut, especially following changes in the indigenous colonic microbiota after antibiotic use (Knight et al., 2015). The *C. difficile* genomes have been well documented with a high proportion of mobile genetic elements and an ultra-low level of genetic conservation (as low as 16%) (Knight et al., 2016), and its phylogenetics and evolutionary clades have been recognized as well (Knight et al., 2015). A whole-genome sequencing (WGS)-based phylogenetic tree showed that *C. difficile* has six main clades (Knight et al., 2015), in which clade 1 is the largest one with a variety of sequence types (STs), including ST2, which usually resulted in mild or moderate *C. difficile* infection (CDI) (Griffiths et al., 2010; Cheng et al., 2016). ST2 exhibits high genetic diversity with various PCR ribotypes (RT) (Griffiths et al., 2010; Jin et al., 2017), two toxin genes (*tcdA* and *tcdB*) are located on a 19.6-kb pathogenicity locus (PaLoc), and no binary toxins are found (Knight et al., 2015). ST2 also belongs to RT014, which is a successful lineage of *C. difficile* as significant reservoirs in both human and porcine populations in Australia, and there were obviously different genomic features and biological phenotypes in ST2 including antibiotic resistance elements and antibiotic resistance phenotype between different resources (Knight et al., 2016). However, the genomic characteristics and biological phenotypes remain to be determined in other regions.

Recent studies showed that the molecular epidemiology of *C. difficile* in China has its own characteristics with specific antibiotic resistance and genotype profiles as below (Tang et al., 2016; Jin et al., 2017; Luo et al., 2019). A meta-analysis displayed that the pooled incidence of toxigenic *C. difficile* in diarrhea patients in Mainland China was approximately 14%, and ST2 was one of the dominant genotypes in China (Tang et al., 2016). However, ST2 usually led to mild or moderate CDI with no other severe symptoms, making it be easily overlooked in clinical treatment (Jin et al., 2017; Luo et al., 2018). Even though antimicrobial resistance phenotypes of *C. difficile* ST2 in Australia have been described as reported previously, there is still a paucity of data on ST2 genomic characteristics and the biological characteristics, including toxin expression and sporulation in other regions.

WGS provides an ultra-fine scale resolution tool for analysis of genomic characteristics, evaluation of bacterial genetic diversity, identification of subtle genetic variability, identification of signatures of clonal transmission, and assessments of *C. difficile* epidemiology of strains implicated in infection recurrences and outbreaks (Didelot et al., 2012; Eyre and Walker, 2013; Martin et al., 2016). Here, a collection of 182 ST2 strains from different regions of human origin with isolation dates between 2011 and 2017 were studied. Genome characteristics associated with virulence and antibiotic resistance genotypes were studied *in silico*, and biological features, including antibiotic resistance phenotypes, toxin expression, and sporulation, were also measured *in vitro*.

## Materials and Methods

### *C. difficile* Isolates and WGS Data

A total of 30 ST2 strains were isolated from CDI patients as part of different CDI surveillance programs conducted in different sites including Zhejiang (*n* = 18) between February 2013 and January 2017, Hebei (*n* = 10) between January 2011 and December 2014, Hunan (*n* = 2) in 2014. The remaining 10 ST2 strains were sourced from CDI patients as part of an international collaboration on molecular characteristics of *C. difficile*. Isolates originated from six sites related to hospitals, including Hong Kong (*n* = 2) in China, Pusan in South Korea (*n* = 1), Fukuoka in Japan (*n* = 1), Singapore (*n* = 3), and Sydney (*n* = 2) and Perth (*n* = 1) in Australia. All the 40 available ST2 isolates with no epidemiological relationships were included in this study by the time the study started. Clinical data were collected after the study was approved by the Ethics Committee of the Hangzhou Medical College. All available genomic data for ST2 strains were included in this study up to January 2017. The genome data of a total of 139 ST2 strains published between 2001 and 2016 were downloaded from the National Center for Biotechnology (NCBI^1^). Raw reads data of 3 ST2 strains (SRR1519431, SRR1519374, and SRR1519422) isolated in 1995 and 1997, respectively, were downloaded from the NCBI database (up to January 2017). The information of the 182 strain was provided in Supplementary Material.

### PCR Ribotyping

Six reference *C. difficile* strains (ATCC 43255, ATCC 43598, BAA-1870, BAA-1803, BAA-1801, and ATCC 700057) were used as controls. PCR ribotyping was performed by using PCR followed by capillary gel electrophoresis described previously (Indra et al., 2008). The 16S rRNA gene primers were labeled at the 5′ end with carboxyfluorescein. After PCR amplification, PCR fragments were analyzed using an ABI 3100 genetic analyzer (Applied Biosystems, Foster City, CA, United States) with a 36-cm capillary loaded with a POP4 gel (Applied Biosystems). The size of each peak was determined using GeneMapper ID-X 1.3 (Applied Biosystems). RT assignment was performed after the data were deposited in the WEBRIBO database^2^.

### Whole-Genome Sequencing and Assembly

Genomic DNA was extracted as previously described (Stabler et al., 2009). WGS libraries were created using TruePrep DNA Library Prep^TM^ Kit V2 (Illumina, Santiago, CA, United States). WGS was performed using the Illumina Hiseq X-ten with 150-base paired-end reads. The sequence data were processed and quality controlled according to a standard pipeline as previously described (Preston et al., 2014). Briefly, FASTQ-formatted sequencing reads were quality controlled with a minimum quality Phred score of 30 (as a rolling average over 4 bases) using trimmomatic v0.36. Trimmomatic v0.36 was also used to remove adapters and low-quality sequences, and 63.051 Gb clean bases were finally generated (1.616 Gb/per isolate, Q20 ≥ 95%) (Bolger et al., 2014). Genome data for the 40 isolates from this study, and the downloaded raw reads for three genomes, were *de novo* assembled using Velvet (version 1.2.10). Optimal *k*-mers fell between 47 and 93 bp, according to the mean value for median contig size of genome assembly (n50). The genomic sequences of the remaining 139 strains were downloaded from the NCBI.

### SNP Detection and Identification

All genome sequences were aligned to the *C. difficile* W0022a reference genome (GCF_002812625.1) and SNPs were identified using MUMmer (v3.23) with default parameters (Kurtz et al., 2004; Li et al., 2016). Following the removal of SNPs within 5 bp of the location interval (less than five bases existed between any two SNPs) by using the perl script (file name: filter_dist.pl.^3^), high-quality SNPs were annotated according to demographic information including clades and locations.

### Recombination Detection and Phylogenetic Analysis

Gubbins was used to detect recombination in newly built whole-genome sequences (Croucher et al., 2015) as previously reported (Knight et al., 2015, 2016), and SNPs located in the recombination regions were identified and removed by using the perl script (file name: gubbinssns.vcf_to_genotype.pl., see text footnote 3). After that, an alignment of non-recombinant SNPs was obtained. A total of 182 *C. difficile* genomes were used to generate core genes by using BLAST with the thresholds of 80% nucleotide sequence identity and 80% of the query length. A total of 1,685 genes were defined as the core gene sets using BLASTp with an E-value of 1e-10, out of which the final SNPs were detected from all non-recombinant SNPs. The Maximum likelihood (ML) tree topology and branch length were inferred using IQ-TREE multicore v1.6.6. The recombination/mutation (*r/m*) ratio was calculated within the deep-branching phylogeny, which gives the relative probability that a nucleotide has changed as a result of recombination relative to a point mutation. Two phylogenetic trees before and after removal of SNPs located in the recombination regions by using Gubbins were compared, and the consistency indexes for two phylogenetic trees were analyzed by using the phangorn package in R (Schliep, 2011).

### Antibiotic Resistance Genes and Virulence Factors

Antibiotic resistance-associated genes were determined using RGI (version 5.1.0) analysis and the CARD antibiotic resistance gene database^4^ (Alcock et al., 2020). Virulence loci were determined by BLAST (Ye et al., 2006; Johnson et al., 2008) analysis of genome sequences in the virulence factors database (VFDB^5^), which aims to provide up-to-date knowledge of virulence factors from various bacteria and serves as a comprehensive warehouse of bacterial pathogenesis knowledge for the scientific community (Liu et al., 2019). The cutoff values were set as 90% nucleotide identity and 90% of the query length for gene coverage to screen virulence loci (Dingle et al., 2019).

### Antibiotic Susceptibility Testing

Twelve antibiotics including vancomycin, metronidazole, moxifloxacin, erythromycin, clindamycin, rifampicin, levofloxacin, gatifloxacin, ciprofloxacin, fusidic acid, tetracycline, and piperacillin-tazobactam (PIP-TAZ) were used to test the minimum inhibition concentration (MIC) of the 40 isolates by the agar dilution method according to standard clinical and laboratory guidelines (CLSI) (Clinical and Laboratory Standards Institute, 2011). The reference strain (ATCC 700057) was included in each test as a control. MIC breakpoints were chosen according to a previous report (Jin et al., 2017).

### Detection of *C. difficile* Toxin B Cytotoxicity and Sporulation Capacity

*Clostridioides difficile* toxin B concentration was quantitatively detected using the real-time cell analysis (RTCA) system according to a previously reported method (Ryder et al., 2010). The toxin B concentration was calculated by a formula which was derived based on the testing results of a panel of purified toxin B standards with known concentrations ranging from 0.02 to 200 ng/μL. The sporulation capacity was measured as reported previously (Qin et al., 2017). A count between 30 and 300 spores on each plate was considered significant.

### Statistical Analysis

Data analysis was performed using Statistical Package for Social Sciences (SPSS, Chicago, IL, United States) version 22.0. The difference in toxin concentration and sporulation capacity was analyzed using a *t*-test or nonparametric test. A *P*-value of <0.05 was considered statistically significant.

## Results

### Genomic Characteristics of *C. difficile* ST2

**FIGURE 1:**
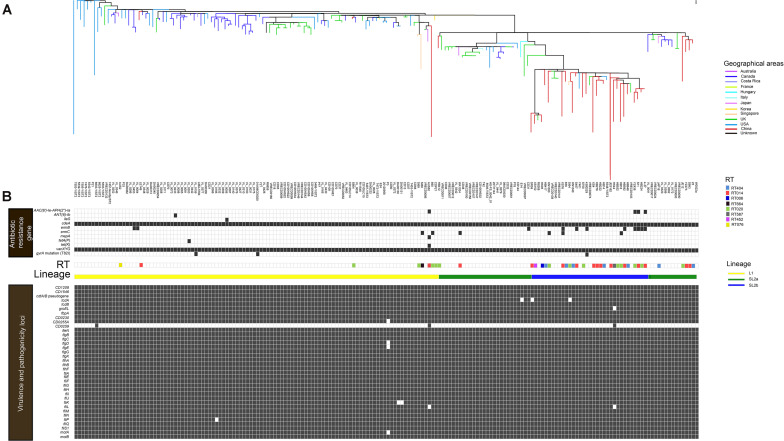
*In silico* predicted antibiotic resistance genes and virulence loci of 182 *C. difficile* ST2 strains. The heat map showed the distribution of antibiotic resistance genes and virulence loci in the genome-wide data. **(A)** The phylogenetic tree. The different colors in the figure represent the different origins. **(B)** Presence is indicated by black bars and absence by white bars. Colored by RT: light blue, RT404; orange, RT014; navy blue, RT006; black, RT664; green, RT020; gray, RT587; rose red, RT452; and yellow, RT076. Sorted by lineage: yellow line, L1; green line, SL2a; and blue line, SL2b.

All 182 WGS data were analyzed after sequence quality control and mapping to the *C. difficile* strain W0022a (NCBI accession: GCF_002812625.1). A total of 14,795 high-quality SNPs were identified after the resulting alignments. Gubbins analysis revealed 10,324 spatially clustered SNPs within 1,352 homologous recombination events. After removing these SNPs, 4,471 high-quality biallelic SNPs were extracted. Of these, 1,249 non-rare (core) SNPs (27.9%, 1,249/4,471) were present in the core genome, which included 966 (77.3%, 966/1,249) non-synonymous SNPs. The *r/m* ratio was approximately 2.31, which was determined through 10,324 SNPs divided by 4,471 SNPs. The phylogenetic tree was explored by high-resolution core genome SNPs analysis as briefly shown in Figure 1A. Firstly, the *tcdB* gene was conserved across all ST2 strains, and 98.4% (179/182) of ST2 had an intact *tcdA* gene, and the remainder carried truncated *tcdA* genes with an in-frame deletion of 1,633, 1,732, and 2,371 bp, respectively. All strains harbored *cdtA/B* pseudogene located in a 4.2-kb pathogenicity locus (CdtLoc) as described (Gerding et al., 2014). The remaining 33 virulence-related genes were distributed in the 182 genomes. Of them, 25 virulence-related genes were found in all ST2 strains. The putative flagellar protein gene (*CD0259*), which was associated with *C. difficile* virulence (Aubry et al., 2012), exists in only three of the ST2 genomes. The remaining seven virulence loci including *groEL*, which plays a role in *C. difficile* cell adherence (Hennequin et al., 2001) and decreases *C. difficile* intestinal colonization (Péchiné et al., 2013), *CD0255A, flgE, fliK, fliL, fliP*, and *motA* were widely distributed in ST2 genomes (Figure 1B).

A total of 10 putative antibiotic resistance genes and one *gyrA* gene variant with amino acid substitution (T82I) were identified within the 182 genomes (Figure 1B). The *vanXY* variant gene, *vanXYG*, mediated aminoglycoside resistance, and the *cdeA* gene, associated with fluoroquinolone resistance, were found in all 182 isolates. The remaining nine genes were found in several genomes as described below. The tetracycline resistance elements, *tetA(P)* and *tet(K)*, were only found in two different *C. difficile* strains (one from Canada, another from Australia) (Figure 1B). For macrolide–lincosamide–streptogramin B (MLS_B_) resistance, the *ermB* and *ermC* genes were scattered in 6.0% (11/182) and 3.3% (6/182) of strains, respectively. Only three strains carried the *gyrA* mutation (T82I). The *AAC(6′)-1e-APH(2^″^)-1a* cassette associated with aminoglycoside resistance was found in 2.2% (4/182) of strains. The above results showed that *C. difficile* ST2 genomes dispersedly harbored a small number of antibiotic resistance genes and associated mutations.

### Phylogenetic Analysis of ST2 Isolates

**FIGURE 2:**
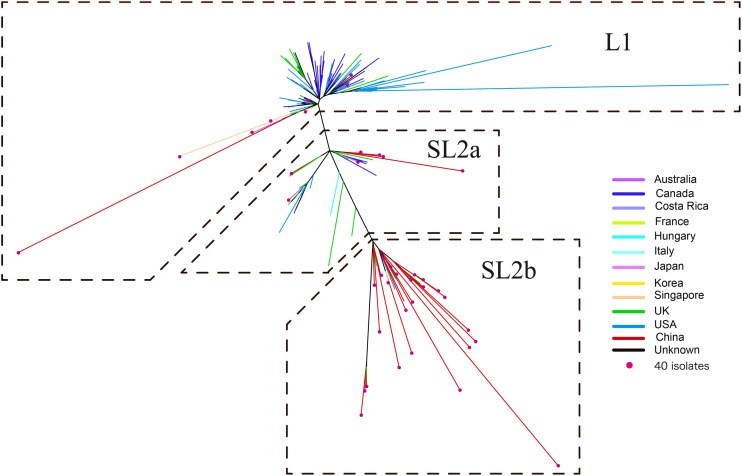
Maximum likelihood phylogeny tree of *C. difficile* ST2. The different colors represented the different origins; the red dot marker was used for 40 *C. difficile* isolates by whole genome sequencing in this study.

A ML phylogeny indicated that the 182 ST2 genomes were obviously divided into two genetically diverse lineages, L1 and L2 (Figure 2), primarily differentiated by 18 unique SNPs (Supplementary Table 1). L2 could be further divided into two sub-lineages, SL2a and SL2b, based on eight specific SNPs (Supplementary Table 2). SL2b primarily comprised most of the *C. difficile* strains from China (25/34, 73.5%), while North America had a high proportion of strains in L1 (66.4%, 71/107) and there was a high diversity of strain sources in SL2a. Various RTs were dispersedly distributed in different sub-lineages as Figure 1B shown. The geographical distribution showed significant differences among L1, SL2a, and SL2b in this ML phylogeny. Interestingly, it has been found that SL2b has only one sublineage-specific non-synonymous mutation, which occurred in the *tcdB* gene (W0022a reference genome, base position: T671,314G; amino acid position: Tyr1975Asp). The *r/m* value of the Chinese SL2b was 2.60, which was higher than those of strains from SL2a (2.33) and SL1 (1.26). There were 28 homologous recombination regions found in all strains belonging to SL2, of which 14 were specific to SL2b; six homologous recombination regions existed in almost all of the strains (Supplementary Figure 1 and Supplementary Table 3).

### *In vitro* Antibiotic Susceptibility Testing

**FIGURE 3:**
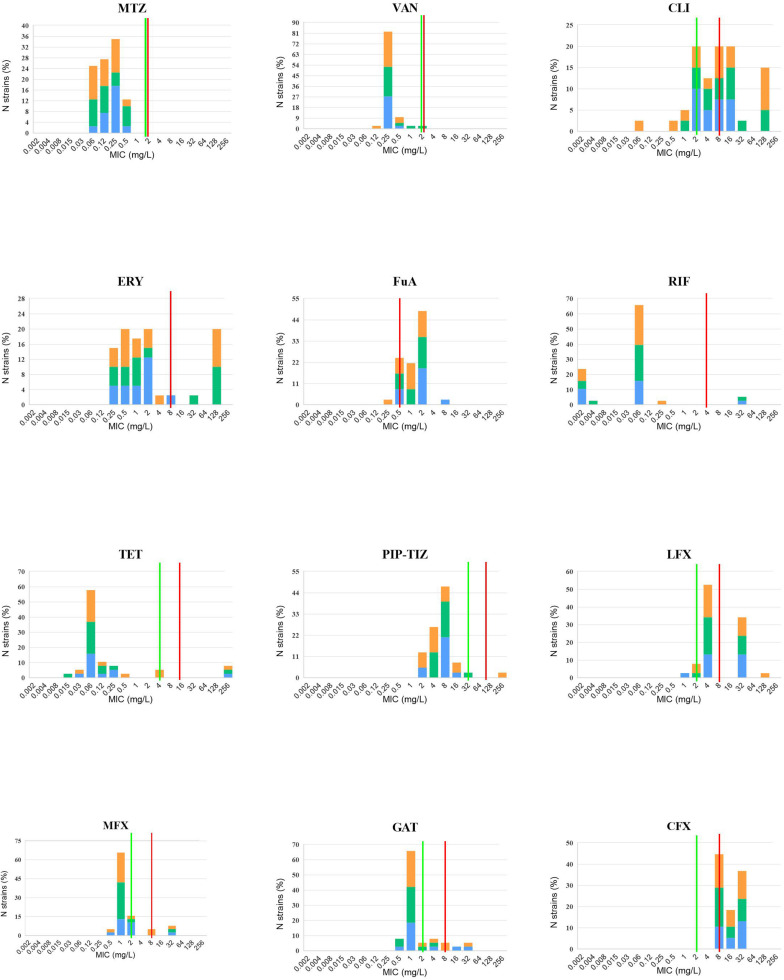
*In vitro* 12 antibiotic susceptibility with susceptible and resistant breakpoints. MIC distributions for 12 antibiotic agents against 40 *C. difficile* isolates, orange: RT014, green: RT020, and blue: RT664, RT006, RT076, RT404, RT452, and RT587. VAN, vancomycin; MTZ, metronidazole; ERY, erythromycin; CLI, Clindamycin; RIF, rifaximin; TET, tetracycline; PIP-TAZ, piperacillin-tazobactam; LFX, levofloxacin; MFX, moxifloxacin; GAT, gatifloxacin; CFX, ciprofloxacin. Where available, established susceptible and resistant breakpoints were indicated by vertical green and red lines, respectively.

Antibiotic susceptibility testing was performed in the 40 isolates sequenced in this study (Table 1). Summary MIC distributions for 12 antibiotic agents, by RT lineage, were presented in Figure 3. The rate of resistance to MLS_B_ was 90.0% (36/40). The rates of resistance to the fourth-generation fluoroquinolones, gatifloxacin (15.0%, 6/40) and moxifloxacin (10.0%, 4/40) were lower than those to the third-generation fluoroquinolones, levofloxacin (82.5%, 33/40) (χ^2^ = 36.47 and 42.29, *P* < 0.001) and ciprofloxacin (92.5%, 37/40) (χ^2^ = 48.32 and 54.48, *P* < 0.001). No isolates were resistant to vancomycin or metronidazole. The rates of resistance to MLS_B_, fluoroquinolone, and fusidic acid (62.5%, 25/40) were distinctly higher than those to rifampicin (5.0%, 2/40) (χ^2^ = 29.57, *P* < 0.001), tetracycline (7.5%, 3/40) (χ^2^ = 26.59, *P* < 0.001), and piperacillin (2.5%, 1/40) (χ^2^ = 32.82, *P* < 0.001). All the RT020 isolates were susceptible to PIP-TAZ, and resistant to MLS_B_ and fluoroquinolone. Of them, 61.5% (8/13), 7.7% (1/13), and 7.7% (1/13) were resistant to fusidic acid, and rifampicin, tetracycline, respectively. All of the RT014 isolates were susceptible to rifampicin; 86.7% (13/15) and 93.3% (14/15) were resistant to fluoroquinolone and MLS_B_, respectively; 60.0% (9/15) were resistant to fusidic acid; 6.7% (1/15) were resistant to tetracycline and piperacillin. The multidrug-resistant (MDR: resistant to ≥3 of these agents) rate was 65.0% (26/40), noted to be high in this study. Even though no significant differences in the antibiotic patterns were found among the different RTs and SLbs, L1 and SL2a, with no strains resistant to rifampin, tetracycline, PIP-TAZ, and moxifloxacin, they obviously presented different resistance patterns from SL2b (Supplementary Table 4).

**TABLE 1:** Clinical information of 40 *C. difficile* ST2 isolate.

Patient characteristics	*N* = 40
**Demographics**	
Age [mean, median (range)] (year)^*a*^	51.0 (3–95)
Gender^*a*^ Male [*n* (%)]	17 (42.5%)
**Isolation place [*n* (%)]**	
** China**	
Zhejiang	18 (45.0%)
Hubei	10 (25.0%)
Hunan	2 (5.0%)
Hong Kong	2 (5.0%)
Japan	1 (2.5%)
South Korea	1 (2.5%)
Singapore	3 (7.5%)
Australia	3 (7.5%)
**Isolation place [*n* (%)]**	
2011–2013	12 (30.0%)
2014	18 (45.0%)
2015–2017	10 (25.0%)
**Ward type [*n* (%)]**	
Gastroenterology	13 (32.5%)
Infectious disease	4 (10.0%)
Oncology	3 (7.5%)
Respiratory	2 (5.0%)
Neurology	2 (5.0%)
Hematology	2 (5.0%)
Geriatrics	1 (2.5%)
Urology	1 (2.5%)
Outpatient	2 (5.0%)
Health Checkup	5 (12.5%)

*^*a*^Information from five strains in Hong Kong and Singapore was not available.*

### Toxin Expression and Sporulation

**FIGURE 4:**
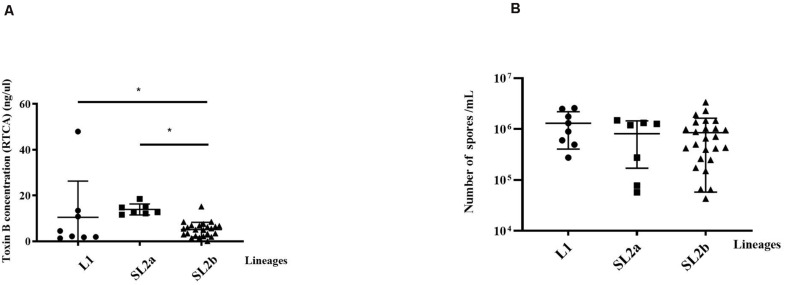
Comparison of toxin production and Sporulation determination in the 40 *C. difficile* ST2 strains with different sub-lineages. **(A)** Toxin B concentrations by the RTCA system; **(B)** sporulation capacity by the heat-induced experiment. The toxin concentrations and sporulation capacity were shown as the mean ± standard deviation. Significant differences were marked with *P < 0.05.

The clinical information for the 40 patients is shown in Table 1. All the 40 ST2 isolates had no epidemiological relationships in spite that some of them belonged to the same RT. Some of the isolates were from the same province but not from the same location. Thus, no same clones existed in these ST2 genomes. The RTCA data showed that the average concentrations of toxin B in groups L1, SL2a, and SL2b were 10.49 ± 15.82, 13.92 ± 2.39, and 5.11 ± 3.20 ng/μL, respectively (Figure 4). *C. difficile* isolates in SL2a had significantly higher toxin concentration abilities than those in SL2b (*t* = 6.709, *P* < 0.001). Spore resuscitation abilities were represented in the mode of InterQuartile Range [M (P25, P75)]. The numbers for *C. difficile* sporulation in L1, SL2a and SL2b were 1.1 × 10^6^ (5.2 × 10^5^, 2.3 × 10^6^)/mL, 1.2 × 10^6^ (7.8 × 10^4^, 1.3 × 10^6^)/mL, and 6.6 × 10^5^ (2.5 × 10^5^, 1.2 × 10^6^)/mL, respectively (Figure 4). There were no statistical differences in sporulation in the three groups (χ^2^ = 2.13, *P* = 0.346).

## Discussion

*Clostridioides difficile* ST2 is one of the most dominant genotypes associated with mild CDI or asymptomatic carriage in China (Tian et al., 2016; Zhang et al., 2016; Jin et al., 2017), whereas the biological phenotypes and genomic characteristics of Chinese ST2 strains remain unexplored thus far. The ST2 lineage might be overshadowed by increasing prevalence of ST37/RT017 worldwide and outbreaks of ST1/RT027 lineage. As the ML phylogenetic trees on ST1/RT027 and ST37/RT017, our data revealed two distinct lineages of *C. difficile* ST2 with multiple independent clonal expansions after removal of SNPs located in the recombination regions. We also compared two phylogeny trees before and after the removal of SNPs located in the recombination regions by using Gubbins. The ML phylogeny tree before removal of SNPs located in the recombination regions showed similarity with SL1, SL2a, and SL2b (data not shown). The results showed that the consistency indexes of the ML phylogenetic trees with and without removal of SNPs located in the recombination regions were 0.9796078 and 0.937613, which was analyzed by using the phangorn package in R, indicating that removal of recombinant SNPs made the remaining SNPs more consistent with the resulting phylogeny. Notably, further analysis indicated that there was a sub-lineage of SL2b divided under L2, most of which (73.5%, 25/34) originated from China, and the average toxin B concentration in SL2b was significantly lower in comparison to those in L1 and SL2a.

ST2 and ST37 are the predominant genotypes in China (Tang et al., 2016; Jin et al., 2017). The T82I gene mutation in *gyrA* found in our study has been reported in the global outbreak of RT027 and RT017 (He et al., 2013; Cairns et al., 2017). The T82I substitution in RT027 and RT017 was globally distributed in both of their two sublineages but was found *in silico* in three ST2 genomes. In addition, a substitution in the *gyrB* gene, also associated with fluoroquinolone resistance, and the substitutions in the rifampin resistance-determining region of the *rpoB* gene (He et al., 2013; Cairns et al., 2017) were not found in ST2 genomes as the RT014 lineage shown (Knight et al., 2016), indicating that ST2 genomes had their own specific molecular characteristics with low diversity of antibiotic resistance-related gene mutations.

The data showed the average toxin production in SL2b was significantly lower than those in L1 and SL2a, and then a sublineage specific non-synonymous mutation in the *tcdB* gene (Y1975D) was found to be located in the C-terminus receptor-binding domain (RBD) of *tcdB*, which is a critical region for interaction with host epithelial cell membranes (Carter et al., 2012; Orth et al., 2014). The demographic information of all 40 isolates was checked, and no epidemiological relationships were found, even in the same RT and the same location. Thus, the ST2 genomes with the mutation of Y1975D were not from the same clone. The *tcdB* gene showed high genetic diversity with classification into eight subtypes (*tcdB*1-8). The subtype *tcdB*1 has the maximum within-subtype variation and consists of three clusters (*tcdB*1a, *tcdB*1b, and *tcdB*1c) as shown in our previous study, and all ST2 strains carried *tcdB*1a (Shen et al., 2020). Most of the *C. difficile* strains from North America, East Asia, and European countries express *tcdB*1, which was closely related to human and animal diseases with more cytotoxicity than other subtypes (Shen et al., 2020). The sequences of RT027-*tcdB*-RBD are genetically divergent from other genotypes, and probably associated with rapid cell entry (Dingle et al., 2011). Although the biological function of the gene mutation (Y1975D) in *tcdB* was unknown as this mutation was not located in the 712bp amplicon previously analyzed (Dingle et al., 2011), the structure of the *tcdB* protein might be impacted, leading to the prevention of toxin B binding to cell surface receptors and decreasing *C. difficile* ST2 cytotoxicity. Thus, we speculated that it was reasonable that CDI led by *C. difficile* ST2 in SL2b had mild or moderate clinical symptoms, and the level of pathological lesion was low, especially for strains in China. However, the mutation in the *tcdB* gene-specific to SL2b from China was not found to be significantly associated with toxin B concentration, probably due to the sampling bias in this study. Thus, more studies should be performed to clarify the relationship between this SNP and low toxin B cytotoxicity and examine its contribution to the decreased ST2 strain virulence.

*Clostridioides difficile* sporulation capacity was associated with bacterial viability, successfully determining the transmission ability of epidemic clones (Qin et al., 2017; Zhu et al., 2018). In this study, no significant difference was found on sporulation capacity of *C. difficile* ST2 between SL2b and other lineages. A previous report provided the first epidemiological evidence on an outbreak of epidemic *C. difficile* genotype ST81 in a general teaching hospital in China (Qin et al., 2017). The data showed that ST81 had similar sporulation capacity in comparison to ST2, but significantly less toxin production than ST2 in China (Qin et al., 2017). Obviously, ST2 strains had a potentially high risk of transmission, leading to a nosocomial outbreak, and the mild clinical symptoms were easily overlooked by clinicians. Moreover, *C. difficile* ST2 with high sporulation capacity might contribute to cross-species transmission from animal to human. Therefore, *C. difficile* ST2 should be closely monitored in future surveillance.

Antibiotic resistance elements are the key factors driving genetic diversity and epidemiological changes in *C. difficile* (Knight et al., 2015). In our study, only two antibiotic resistance loci (*cdeA* and *vanXYG*) existed in all ST2 strains and other antibiotic resistance loci were dispersedly distributed in ST genomes. Similar data were also presented in the previous study on RT014 lineage, mainly including ST2, ST13, and ST49 (Knight et al., 2016). In comparison to other STs in RT014, the ST2 genome has fewer antibiotic resistance genes and associated SNPs. The previous study also showed that only one ST2 strain simultaneously presented phenotypic resistance to tetracycline and MLS_B_ with positivity for *tetM* and *ermB*, and ST2, except two strains that were resistant to aminoglycoside-streptothricin, but without any resistance cassettes (Knight et al., 2016). Similarly, our data showed that only two strains carried *tetA(P)* and *tet(K)*, and two aminoglycoside-streptothricin resistance cassettes [*AAC(6′)-1e-APH(2^″^)-1a* and *ANT(6)-1b*] existed in each of five strains. However, *ermB* was present in the 11 strains, of which 83.3% (10/12) were from SL2b. The rates of resistance to the third-generation fluoroquinolones were significantly higher than those to the fourth-generation fluoroquinolones. According to *in silico* antibiotic resistance analysis, the T82I SNP in *gyrA* was only found in three strains, but *cdeA* existed in all ST2 strains. Therefore, we predicted that the *cdeA* gene was the main reason for third-generation fluoroquinolones resistance in *C. difficile* ST2, and but did not mediate resistance to fourth-generation fluoroquinolones. Based on the MIC data reported here, only 30.6% (11/36) of MLS_B_ resistant isolates contained the *ermB* or *ermC* gene, suggesting that these antibiotic resistance genes alone did not always lead to phenotypic resistance, or other alternative antibiotic resistance mechanisms might exist (Knight et al., 2019). We did not find vancomycin-resistant strains in this study despite finding the *vanXYG* gene in all 182 isolates, demonstrating that *in silico* vancomycin genotyping was a poor predictor of vancomycin phenotype in *C. difficile*.

There is still a paucity of antibiotic resistance data for *C. difficile* ST2 strains in China. In this study, we found all ST2 isolates susceptible to the first-line human CDI therapies vancomycin and metronidazole. The antibiotic resistance pattern of *C. difficile* ST2 observed in this study differed dramatically from that in our previous studies (Jin et al., 2017). The resistance rate for clindamycin (17/18, 94.4%) was markedly higher than that reported previously, but that for fusidic acid, erythromycin, rifampicin, and tetracycline were lower than in previous studies (Jin et al., 2017). The antibiotic resistance pattern in ST strains observed in this study also differed dramatically from that in a systematic review and meta-analysis in China in 2016 (Tang et al., 2016), in which data on erythromycin, clindamycin, and rifampicin showed higher resistance rates than those in ST2, but our data reported here shows high rates of resistance to fusidic acid and levofloxacin in comparison to the systematic review data (Tang et al., 2016). However, the above review data did not provide detailed information about RTs, thus more data on antibiotic resistance specific to *C. difficile* ST2 should be investigated in future studies.

Our study had some limitations as described below. Firstly, the number of isolates investigated (*n* = 40) was low relative to the phylogenetic analysis of ST2 genomes, and the number of strains from other regions or countries is limited. Thus, greater numbers of strains from various regions in China and other countries would enhance the understanding of the phylogenetic analysis of ST2 genomes. Secondly, clinical information and biological features from the other 142 strains were unavailable, including RT types and phenotypic antibiotic resistance. The antibiotic resistance phenotypic-genotypic concordance was still unclear in *C. difficile* ST2. We are going to collecting more ST2 strains from different provinces in China and other countries in order to confirm the biological features of ST2 strains in SL2b in the near future.

## Conclusion

Our study revealed two distinct lineages in *C. difficile* ST2 genomes with many virulence loci and few antibiotic resistance elements. SL2b was exclusively identified with a sub-lineage-dependent genome mutation (Y1975D) in *tcdB*, mainly in *C. difficile* strains from China expressing low toxin B, which might be associated with mild or moderate CDI.

## Data Availability Statement

The original contributions presented in the study are publicly available. The genomic data of the 39 isolates sequenced in this study were deposited in the NCBI database under study accession number PRJNA591265. The accession number of *C. difficile* W0022a is GCF_002812625.1.

## Ethics Statement

The studies involving human participants were reviewed and approved by the Hangzhou Medical College. Written informed consent to participate in this study was provided by the participants’ legal guardian/next of kin.

## Author Contributions

DJ, Y-WT, and XW conceived the study, designed the experiments, and revised the manuscript. XX, QB, and XS collected the samples and performed the experiments. XX, YL, QB, QL, and MW analyzed the data. QB, SL, BZ, and GY performed the statistical analysis. XX, YL, and QB drafted the manuscript. DJ, LC, and Y-WT supervised the study. All authors edited and approved the final version of the manuscript. All corresponding authors had full access to all the data in this study and had final responsibility for the decision to submit for publication.

## Conflict of Interest

The authors declare that the research was conducted in the absence of any commercial or financial relationships that could be construed as a potential conflict of interest.

## Footnotes

1 https://www.ncbi.nlm.nih.gov

2 https://webribo.ages.at/

3 https://github.com/ChaseFor/Gubbin-snp

4 https://card.mcmaster.ca/home

5 http://www.mgc.ac.cn/VFs/main.htm
