# Supplementary material for: Comparative Whole Genome Sequence Analysis and Biological Features of Clostridioides difficile Sequence Type 2‡
Source: Front Microbiol. 2021 Jul 5;12:651520. doi: 10.3389/fmicb.2021.651520 (PMC8287029; doi:10.3389/fmicb.2021.651520)
Supplement: Supplementary file 2 [file Data_Sheet_1.docx]

**Figure Legend**

**Fig. S1.** The strains were sorted according to ML-tree as Fig.2 shown. A: the W0022a reference genome sequence. B: heatmap (centre): density of recombination events detected within the ST2 genomes. Red represented the recombination region, and 14 specific Homologous recombination regions were found in the black dotted frame.

**
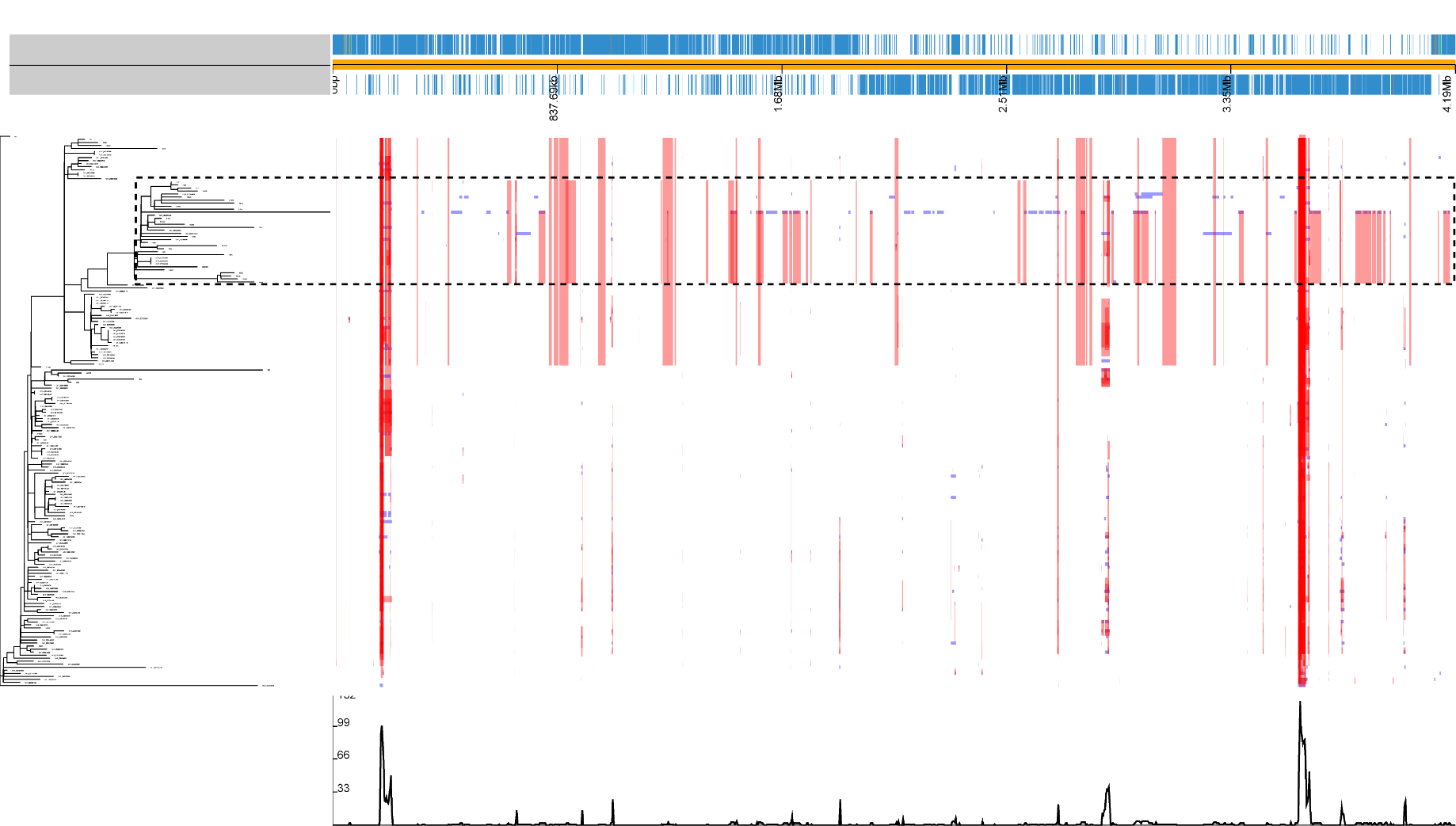
**

B

A

Fig. S1. The recombination regions of the 182 *C. difficile* ST2 strains

Table S1. The eighteen SL-defining SNPs

| SL | Position^a^ | R^b^ | A^c^ | Amino Acid | Codon mutate | AA mutate | Mutate Type | Number of  isolates with SNP | Gene | Describe |
| --- | --- | --- | --- | --- | --- | --- | --- | --- | --- | --- |
| L1 | 3114547 | T | G | 198 | TCA<->GCA | S<->A | nonsynonymous | 105 | *CWR55_RS14560* | nitrilase |
| L1 | 831837 | A | G | 557 | ATA<->ATG | I<->M | nonsynonymous | 97 | *CWR55_RS04040* | penicillin-binding transpeptidase |
| SL2a | 1105064 | T | C | 178 | TTT<->CTT | F<->L | nonsynonymous | 22 | *CWR55_RS05240* | membrane protein |
| SL2a | 1633214 | A | G | 242 | GAA<->GAG | E<->E | synonymous | 22 | *CWR55_RS07725* | methionyl aminopeptidase |
| SL2a | 2212046 | G | A | 50 | GCA<->ACA | A<->T | nonsynonymous | 22 | *CWR55_RS10525* | glutamyl aminopeptidase |
| SL2a | 2358100 | G | A | 266 | GTA<->ATA | V<->I | nonsynonymous | 22 | *CWR55_RS11250* | VWA domain-containing protein |
| SL2a | 3213977 | A | G | 118 | TCA<->TCG | S<->S | synonymous | 22 | *CWR55_RS14985* | aliphatic sulfonate ABC transporter  substrate-binding protein |
| SL2a | 3896061 | G | A | 111 | GCC<->ACC | A<->T | nonsynonymous | 22 | *CWR55_RS18170* | polysaccharide biosynthesis protein |
| SL2a | 636368 | C | T | 187 | CTG<->TTG | L<->L | synonymous | 21 | *CWR55_RS03255* | alpha/beta hydrolase |
| SL2a | 1174147 | C | T | 170 | GCC<->GTC | A<->V | nonsynonymous | 21 | *CWR55_RS05580* | transcription factor FapR |
| SL2b | 1781153 | A | G | 329 | AAA<->AAG | K<->K | synonymous | 34 | *CWR55_RS08420* | DUF917 domain-containing protein |
| SL2b | 2529549 | A | G | 166 | GAA<->GAG | E<->E | synonymous | 34 | *CWR55_RS12080* | efflux RND transporter periplasmic adaptor subunit |
| SL2b | 2530215 | C | T | 388 | ATC<->ATT | I<->I | synonymous | 34 | *CWR55_RS12080* | efflux RND transporter periplasmic adaptor subunit |
| SL2b | 2553202 | T | C | 41 | ATT<->ATC | I<->I | synonymous | 34 | *CWR55_RS12165* | MFS transporter |
| SL2b | 2574144 | G | A | 128 | GAG<->GAA | E<->E | synonymous | 34 | *raiA* | ribosomal subunit interface protein |
| SL2b | 2575448 | T | C | 108 | TCT<->TCC | S<->S | synonymous | 34 | *CWR55_RS12290* | GatB/YqeY domain-containing protein |
| SL2b | 2576700 | T | C | 379 | TTG<->CTG | L<->L | synonymous | 34 | *CWR55_RS12305* | [N(6)-L-threonylcarbamoyladenosine (37)-C(2)]-methylthiotransferase MtaB |
| SL2b | 671314 | T | G | 1975 | TAT<->GAT | Y<->D | nonsynonymous | 33 | *tcdB* | glycosylating toxin TcdB |

^a^ Positions refer to those in the W0022a genome; ^b^ Reference base; ^c^ Alternative base

Table S2. The eight SL2b-defining SNPs

| Position^a^ | R^a^ | A^b^ | Amino Acid | Codon mutate | AA mutate | Mutate Type | Number of isolates with SNP | Gene | Describe |
| --- | --- | --- | --- | --- | --- | --- | --- | --- | --- |
| 1781153 | A | G | 329 | AAA<->AAG | K<->K | synonymous | 34 | *CWR55_RS08420* | DUF917 domain-containing protein |
| 2529549 | A | G | 166 | GAA<->GAG | E<->E | synonymous | 34 | *CWR55_RS12080* | efflux RND transporter periplasmic adaptor subunit |
| 2530215 | C | T | 388 | ATC<->ATT | I<->I | synonymous | 34 | *CWR55_RS12080* | efflux RND transporter periplasmic adaptor subunit |
| 2553202 | T | C | 41 | ATT<->ATC | I<->I | synonymous | 34 | *CWR55_RS12165* | MFS transporter |
| 2574144 | G | A | 128 | GAG<->GAA | E<->E | synonymous | 34 | *raiA* | ribosomal subunit interface protein |
| 2575448 | T | C | 108 | TCT<->TCC | S<->S | synonymous | 34 | *CWR55_RS12290* | GatB/YqeY domain-containing protein |
| 2576700 | T | C | 379 | TTG<->CTG | L<->L | synonymous | 34 | *CWR55_RS12305* | [N(6)-L-threonylcarbamoyladenosine(37)-C(2)]-methylthiotransferase MtaB |
| 671314 | T | G | 1975 | TAT<->GAT | Y<->D | nonsynonymous | 33 | *tcdB* | glycosylating toxin TcdB |

^a^ Positions refer to those in the W0022a genome; ^b^: reference base; ^c^: alternative base

Table S3. Part of specific homologous recombination of *C. difficile* ST2 strains

| Region^a^ start | Region end | numbers of strain | numbers of strain in L1 | numbers of strain in SL2a | numbers of strain in SL2b | Specific to lineage |
| --- | --- | --- | --- | --- | --- | --- |
| 3098314 | 3149706 | 75 | 0 | 41 | 34 | SL2 |
| 846515 | 880314 | 75 | 0 | 41 | 34 | SL2 |
| 1232927 | 1269279 | 75 | 0 | 41 | 34 | SL2 |
| 2772595 | 2809176 | 75 | 0 | 41 | 34 | SL2 |
| 989513 | 1020245 | 75 | 0 | 41 | 34 | SL2 |
| 3603003 | 3610538 | 75 | 0 | 41 | 34 | SL2 |
| 3482954 | 3490267 | 75 | 0 | 41 | 34 | SL2 |
| 1277076 | 1281175 | 75 | 0 | 41 | 34 | SL2 |
| 4019153 | 4025276 | 75 | 0 | 41 | 34 | SL2 |
| 3286655 | 3296318 | 75 | 0 | 41 | 34 | SL2 |
| 176475 | 186594 | 75 | 0 | 41 | 34 | SL2 |
| 1589041 | 1597502 | 75 | 0 | 41 | 34 | SL2 |
| 824897 | 843776 | 75 | 0 | 41 | 34 | SL2 |
| 198877 | 202163 | 75 | 0 | 41 | 34 | SL2 |
| 3620505 | 3629903 | 75 | 0 | 41 | 34 | SL2 |
| 2825053 | 2836002 | 75 | 0 | 41 | 34 | SL2 |
| 208761 | 219728 | 75 | 0 | 41 | 34 | SL2 |
| 808168 | 819542 | 75 | 0 | 41 | 34 | SL2 |
| 2098229 | 2110076 | 75 | 0 | 41 | 34 | SL2 |
| 3004144 | 3012182 | 75 | 0 | 41 | 34 | SL2 |
| 3643320 | 3645903 | 75 | 0 | 41 | 34 | SL2 |
| 3324141 | 3325951 | 75 | 0 | 41 | 34 | SL2 |
| 1503733 | 1508420 | 75 | 0 | 41 | 34 | SL2 |
| 430484 | 434394 | 75 | 0 | 41 | 34 | SL2 |
| 926004 | 926804 | 75 | 0 | 41 | 34 | SL2 |
| 2812124 | 2814897 | 75 | 0 | 41 | 34 | SL2 |
| 976894 | 977899 | 75 | 0 | 41 | 34 | SL2 |
| 316129 | 320412 | 75 | 0 | 41 | 34 | SL2 |
| 2891559 | 2900738 | 34 | 0 | 0 | 34 | SL2b |
| 2577502 | 2585691 | 34 | 0 | 0 | 34 | SL2b |
| 1477466 | 1497786 | 34 | 0 | 0 | 34 | SL2b |
| 871321 | 909574 | 34 | 0 | 0 | 34 | SL2b |
| 653011 | 668644 | 34 | 0 | 0 | 34 | SL2b |
| 2878100 | 2880729 | 34 | 0 | 0 | 34 | SL2b |
| 2554982 | 2565874 | 34 | 0 | 0 | 34 | SL2b |
| 1952254 | 1955860 | 34 | 0 | 0 | 34 | SL2b |
| 1226610 | 1229312 | 34 | 0 | 0 | 34 | SL2b |
| 681650 | 687727 | 34 | 0 | 0 | 34 | SL2b |
| 1395691 | 1399905 | 34 | 0 | 0 | 34 | SL2b |
| 1783570 | 1788598 | 34 | 0 | 0 | 34 | SL2b |
| 3760935 | 3761118 | 34 | 0 | 0 | 34 | SL2b |
| 1506191 | 1511587 | 34 | 0 | 0 | 34 | SL2b |
| 3604302 | 3614730 | 176 | 101 | 41 | 34 | common |
| 3629492 | 3632672 | 176 | 101 | 41 | 34 | common |
| 3606464 | 3614730 | 178 | 103 | 41 | 34 | common |
| 3629492 | 3632588 | 178 | 103 | 41 | 34 | common |
| 3629504 | 3632588 | 179 | 104 | 41 | 34 | common |
| 3605258 | 3611636 | 179 | 104 | 41 | 34 | common |

^a^ Positions refer to those in the W0022a reference genome.

Table S4. Correlations among PCR ribotypes, SL and antimicrobial susceptibility patterns of *C. difficile* ST2 isolates

| Antimicrobial agents | No. (%) non-susceptible isolates | | | | | | | | | |
| --- | --- | --- | --- | --- | --- | --- | --- | --- | --- | --- |
|  | Total isolates  (n = 40) | MLST type | | Results of analysis | | PCR ribotype | | | Results of analysis | |
|  |  | SL2b  (n = 25) | Other SLs  (n = 15) | χ^2^ | *P* value | 020  (n =13) | 014  (n = 15) | Others  (n = 12) | χ^2^ | *P* value |
| Metronidazole | 0 | 0 | 0 | - | - | 0 | 0 | 0 | - | - |
| Vancomycin | 0 | 0 | 0 | - | - | 0 | 0 | 0 | - | - |
| Clindamycin | 34 (85.0) | 20 (80.0) | 14 (93.3) | 1.307 | 0.253 | 12 (92.3) | 14 (93.3) | 8 (66.7) | 4.525 | 0.104 |
| Erythromycin | 10 (25.0) | 8 (32.0) | 2 (13.3) | 1.742 | 0.187 | 5 (38.5) | 4 (26.7) | 1 (8.3) | 3.056 | 0.217 |
| Fusidic acid | 25 (62.5) | 16 (64.0) | 9 (60.0) | 0.064 | 0.800 | 8 (61.5) | 9 (60.0) | 8 (66.7) | 0.134 | 0.935 |
| Rifampin | 2 (5.0) | 2 (8.0) | 0 | 1.263 | 0.261 | 1 (7.7) | 0 | 1(8.3) | 1.269 | 0.530 |
| Tetracycline | 3 (7.5) | 3 (12.0) | 0 | 1.946 | 0.163 | 1 (7.7) | 1 (6.7) | 1 (8.3) | 0.028 | 0.986 |
| PIP-TAZ | 1(2.5) | 1(4.0) | 0 | 0.615 | 0.433 | 0 | 1 (6.7) | 0 | 1.709 | 0.425 |
| Levofloxacin | 33 (82.5) | 20 (80.0) | 13 (86.7) | 0.289 | 0.591 | 12 (92.3) | 11 (73.3) | 10 (83.3) | 1.745 | 0.418 |
| Moxifloxacin | 4 (10.0) | 4 (16.0) | 0 | 2.667 | 0.102 | 1 (7.7) | 2 (13.3) | 1 (8.3) | 0.299 | 0.861 |
| Gatifloxacin | 6 (15.0) | 4 (16.0) | 2 (13.3) | 0.052 | 0.819 | 1 (7.7) | 3 (20.0) | 2 (16.7) | 0.865 | 0.649 |
| Ciprofloxacin | 37 (92.5) | 23 (92.0) | 14 93.3) | 0.024 | 0.877 | 13(100.0) | 13 (86.7) | 11 (91.7) | 1.802 | 0.406 |
| MDR | 26 (65.0) | 17 (68.0) | 9 (60.0) | 0.264 | 0.608 | 10 (76.9) | 10 (66.7) | 6 (50.0) | 2.017 | 0.365 |
